# Informing Decision-making for Transected Margin Reresection in Intraductal Papillary Mucinous Neoplasm-derived Pancreatic Cancer: An International Multicenter Study

**DOI:** 10.1097/SLA.0000000000006532

**Published:** 2024-09-12

**Authors:** Joseph R. Habib, Ingmar F. Rompen, Benedict Kinny-Köster, Brady A. Campbell, Paul C.M. Andel, Greg D. Sacks, Adrian T. Billeter, Hjalmar C. van Santvoort, Lois A. Daamen, Ammar A. Javed, Beat P. Müller-Stich, Marc G. Besselink, Markus W. Büchler, Jin He, Christopher L. Wolfgang, I. Quintus Molenaar, Martin Loos

**Affiliations:** * Department of Surgery, New York University Langone Health, New York, NY; † Department of Surgery, Regional Academic Cancer Center Utrecht, UMC Utrecht Cancer Center & St. Antonius Hospital Nieuwegein, Utrecht, The Netherlands; ‡ Department of General, Visceral, and Transplantation Surgery, Heidelberg University Hospital, Heidelberg, Germany; § Department of Surgery, Amsterdam UMC, University of Amsterdam, Amsterdam, The Netherlands; ‖ Cancer Center Amsterdam, The Netherlands; ¶ Department of Surgery, Johns Hopkins Hospital, Baltimore, MD; # Clarunis University Digestive Health Care Center Basel, Basel, Switzerland; ** Division of Imaging and Oncology, University Medical Center Utrecht, Utrecht, The Netherlands; †† Department of Pancreatic Surgery, Champalimaud Foundation, Lisbon, Portugal

**Keywords:** intraductal papillary mucinous neoplasm, pancreatic neoplasms, pancreatic cyst, pancreatic cancer, margin, R1, dysplasia

## Abstract

**Objective:**

To assess the prognostic impact of margin status in patients with resected intraductal papillary mucinous neoplasms (IPMN)-derived pancreatic ductal adenocarcinoma (PDAC) and to inform future intraoperative decision-making on handling differing degrees of dysplasia on frozen section.

**Background:**

The ideal oncologic surgical outcome is a negative transection margin with normal pancreatic epithelium left behind. However, the prognostic significance of reresecting certain degrees of dysplasia or invasive cancer at the pancreatic neck margin during pancreatectomy for IPMN-derived PDAC is debatable.

**Methods:**

Consecutive patients with resected and histologically confirmed IPMN-derived PDAC (2002–2022) from 6 international high-volume centers were included. The prognostic relevance of a positive resection margin (R1) and degrees of dysplasia at the pancreatic neck margin were assessed by log-rank test and multivariable Cox-regression for overall survival (OS) and recurrence-free survival.

**Results:**

Overall, 832 patients with IPMN-derived PDAC were included, with 322 patients (39%) having an R1-resection on final pathology. Median OS (mOS) was significantly longer in patients with an R0 status compared with those with an R1 status (65.8 vs 26.3 mo *P*<0.001). Patients without dysplasia at the pancreatic neck margin had similar OS compared with those with low-grade dysplasia (mOS: 78.8 vs 66.8 mo, *P*=0.344). However, high-grade dysplasia (mOS: 26.1 mo, *P*=0.001) and invasive cancer (mOS: 25.0 mo, *P*<0.001) were associated with significantly worse OS compared with no or low-grade dysplasia. Patients who underwent conversion of high-risk margins (high-grade or invasive cancer) to a low-risk margin (low-grade or no dysplasia) after intraoperative frozen section had significantly superior OS compared with those with a high-risk neck margin on final pathology (mOS: 76.9 vs 26.1 mo *P*<0.001).

**Conclusions:**

In IPMN-derived PDAC, normal epithelium or low-grade dysplasia at the neck have similar outcomes, while pancreatic neck margins with high-grade dysplasia or invasive cancer are associated with poorer outcomes. Conversion of a high-risk to low-risk margin after intraoperative frozen section is associated with survival benefit and should be performed when feasible.

It is estimated that almost 1 of every 5 pancreatic cancers arise from intraductal papillary mucinous neoplasms (IPMN).^1^ These precursor lesions display varying degrees of cellular atypia ranging from low-grade (LGD) to high-grade dysplasia (HGD) and eventual progression to invasive carcinoma.^2,3^ The ideal time to operate on patients with an IPMN is when HGD is present and before the progression to invasive carcinoma. However, when resected at an invasive stage, local and systemic disease recurrence dictate survival prognosis.^4,5^ Even after resection, a notable proportion of patients with resected low-grade or high-grade IPMNs are at a risk of developing invasive cancer years after surgery.^6^ Therefore, the best chance for cure for IPMN-derived pancreatic ductal adenocarcinoma (PDAC) is a complete surgical resection of both invasive and noninvasive components.^7^

At the time of histopathologic assessment of the surgical specimen, the resection margin can be negative (true R0), have a degree of dysplasia (low-grade or high-grade), or have invasive carcinoma at (direct R1) or within 1 mm (indirect R1) of the margin. Among patients with resected IPMN-derived PDAC, HGD or invasive carcinoma at the margin has been associated with disease recurrence and worse survival.^8^ Nevertheless, the implementation of frozen sections to assess transected margins intraoperatively is not yet universally adopted.^9^ Although the ideal surgical outcome is a negative transection margin with normal pancreatic epithelium, the prognostic significance of reresecting positive margins in traditional pancreatic intraepithelial neoplasm (PanIN)-derived PDAC remains debatable with conflicting data.^10–13^ Evidence for intraoperative margin handling and the benefit of reresection after frozen sectioning in IPMNs is even less developed.^8,14,15^ Although guidelines recommend reresection of margins with HGD or invasive cancer, some studies have suggested that the presence of invasive cancer associated with the IPMN in the primary specimen will drive outcomes. Thus, reresection of HGD at the margin in these patients may not have an associated survival benefit.^16–19^

The present study aims to provide a comprehensive report and assessment of the prognostic impact of margin status in patients with resected IPMN-derived PDAC and to inform future intraoperative decision-making on handling differing degrees of dysplasia observed during intraoperative frozen sectioning.

## METHODS

Consecutive patients who underwent resection and had histologically confirmed IPMN-derived PDAC between 2002 and 2022 from 6 international high-volume pancreas surgery centers, including University Hospital of Basel, Amsterdam University Medical Center, Heidelberg University Hospital, Johns Hopkins Hospital, New York University Langone Health, and the Regional Academic Cancer Center Utrecht were screened for eligibility. Patients with postoperative mortality (within 90 days of index operation), concomitant PanIN-derived PDAC, or grossly positive resection margins (R2) were excluded. Institutional review board approval was obtained, and the study was compliant with Health Insurance Portability and Accountability Act regulations. The study was performed in compliance with the strengthening the reporting of observational studies in epidemiology (STROBE) guidelines.^20^

Histopathologic evaluations of resected specimens were performed by trained pathologists at each institution. All included patients were verified to have invasive cancer arising in association with the IPMN.

The resection margin was defined as R1 in cases with microscopic evidence of invasive cancer within 1 millimeter and further stratified as direct when invasive cells were present at the margin (direct R1) or indirect when within 1 mm but not directly involving the resection margin. The R0 margins were further substratified as normal pancreatic epithelium (true R0), LGD, or HGD within 1 mm of the resection margin. In cases of different degrees of dysplasia or carcinoma being present within the margin or at multiple margin sites, the worst grade was used for analysis. Carbohydrate antigen 19-9 (CA19-9) was defined as normal (5–37 U/mL), elevated (>37 U/mL) or nonsecretor (<5 U/m:L). The eighth edition of the American Joint Committee on Cancer (AJCC)^21^ T-stage (size of invasive component) criteria and N-stage was used and T3 and T4 tumors were combined into 1 group.^17,22,23^ Overall survival (OS) was calculated as the time between date of surgery and date of death. Recurrence-free survival (RFS) was defined as the time between the date of surgery and date of recurrence or death. Patients were censored at the date of last follow-up for patients still alive or without recurrence, respectively.

First, all patients were stratified into R1 and R0 resection groups. Baseline characteristics and perioperative and longitudinal outcomes (OS and RFS) were compared. Recurrence rates based on the location of R1 margin on final pathology were described and the prognostic impact of direct versus indirect R1 margins was evaluated. Patients with an R0 resection were then substratified into true R0, LGD, and HGD groups and time to event outcomes were compared with each other and to R1 resections. Following this, the impact of intraoperative frozen sectioning of the transected neck margin was examined. We investigated its impact on intraoperative decision-making in terms of extended resections and the final pathologic neck margin status, including all patients with available data, regardless of other margin status. Next, a subgroup analysis was performed on patients who underwent intraoperative frozen sectioning of the pancreatic neck (a surgically modifiable margin) and excluded patients with a preoperative planned total pancreatectomy. This subanalysis only included patients where the pancreatic neck was the worst pathologic margin, ie, if another margin had worse pathology, those patients were excluded from this analysis. In this cohort, the impact of margin reresection of a high-risk margin (HGD or invasive cancer) and conversion to a low-risk margin (true R0 or LGD) on survival was assessed. Patients with a converted high-risk to low-risk neck margin were compared with patients with a high-risk neck margin on final pathology. An additional multivariable Cox-regression adjusting for T-stage and N-stage was performed to determine the adjusted impact of margin reresection and conversion. A final analysis, comparing patients with a converted high-risk to low-risk neck margin and patients with a high-risk neck margin on final pathology, regardless of alternative margin site status was performed, given that alternative margin status is not known intraoperatively.

Baseline characteristics and clinicopathologic data were displayed as categorical variables with frequencies and percentages. These were compared using a χ^2^ test. Kaplan-Meier survival curves were used for visualizing all time-to-event analyses and to derive median OS (mOS), RFS (mRFS), and time to local progression with 95% CIs. A Log-rank test was used for all unadjusted survival comparisons. Multivariable Cox-regression was performed to estimate hazard ratios (HR) for the association between high-risk margin and OS and RFS adjusting for age, CA19-9, operation, grade of differentiation, T-stage, N-stage, perineural invasion, lymphovascular invasion, and adjuvant chemotherapy. A *P* value <0.05 was used to define statistical significance. Statistical analysis was performed using the “R” statistical software (version 4.2.3) using “survminer,” “survival,” and “ggplot2” packages.

## RESULTS

### Initial Study Population Stratified by R1 and R0 Margin Status on Final Pathology

Overall, 832 patients with IPMN-derived PDAC were included. Of these, mean age was 69.3 years (SD ±9.7) and 401 patients (48%) were male. R1 status was identified in 322 patients (39%) on final pathology. Compared with patients with an R0 status, patients with an R1 resection were more likely to have an elevated CA19-9 (*P*<0.001), advanced T-stage (*P*<0.001), advanced N-stage (*P*<0.001), poorer grade of differentiation (*P*<0.001), presence of lymphovascular and perineural invasion (*P*<0.001), and tubular histology (*P*<0.001). Further clinicopathologic characteristics are summarized in Table 1. Perioperative outcomes are summarized in Supplemental Text 1, Supplemental Digital Content 1, http://links.lww.com/SLA/F302. The R1 margin rate on final pathology between 2002 and 2011 was 41%, which was comparable to 38% from 2012 to 2022 (*P*=0.372).

**TABLE 1:** Baseline Demographics and Clinicopathologic Characteristics for the Study Population Stratified by Margin Status

Variable	All N=832	R0 margin N=510	R1 margin N=322	*P*
Age, mean±SD	69.34±9.69	69.68±9.68	68.81±9.69	0.200
Male, n (%)	401 (48)	243 (48)	158 (49)	0.689
CA19-9 (U/mL), n (%)				
Normal	242 (34)	169 (40)	73 (24)	**<0.001**
Elevated	425 (59)	216 (51)	209 (70)	—
Non-secreter	51 (7)	34 (8)	17 (6)	—
Unknown	114	91	23	—
Neoadjuvant Chemotherapy, n (%)	45 (5)	23 (5)	22 (7)	0.149
Year of operation, n (%)	—	—	—	0.740
2002–2010	264 (32)	164 (32)	100 (31)	—
2011–2022	568 (68)	346 (68)	222 (69)	—
Operation, n (%)				0.082
Pancreatoduodenectomy	444 (53)	280 (55)	164 (51)	—
Distal pancreatectomy	165 (20)	107 (21)	58 (18)	—
Total pancreatectomy	223 (27)	123 (24)	100 (31)	—
T stage, n (%)	—	—	—	**<0.001**
pT1	228 (28)	213 (42)	15 (5)	—
pT2	225 (27)	141 (28)	84 (26)	
pT3-4	376 (45)	154 (30)	222 (69)	—
Unknown	3	2	1	—
N stage, n (%)	—	—	—	**<0.001**
pN0	401 (48)	324 (64)	77 (24)	—
pN1	233 (28)	117 (23)	116 (36)	—
pN2	196 (24)	68 (13)	128 (40)	—
Unknown	2	1	1	—
Grade of differentiation, n (%)	—	—	—	**<0.001**
Well	82 (11)	80 (17)	2 (1)	—
Moderate	465 (60)	278 (59)	187 (62)	—
Poor	229 (30)	117 (25)	112 (37)	—
Unknown	56	35	21	—
Lymphovascular invasion, n (%)	250 (43)	119 (31)	131 (66)	**<0.001**
Perineural invasion, n (%)	398 (63)	205 (52)	193 (81)	**<0.001**
Tubular histology, n (%)	542 (84)	327 (78)	215 (93)	**<0.001**

### R1 Margin Site Location on Final Pathology and Rate of Recurrence

Of patients with an R1 margin on final pathology and known location, 31 (16%) had an isolated positive margin at the pancreatic neck, while 53 (28%) had an isolated positive margin at the uncinate process. Multiple R1 sites were present in 27 patients (14%). A more detailed R1 breakdown with respective local recurrence rates per margin site is summarized in Supplemental Table 1, Supplemental Digital Content 1, http://links.lww.com/SLA/F302.

### Longitudinal and Oncologic Outcomes Based on R1 and R0 Margin Status on Final Pathology

**FIGURE 1:**
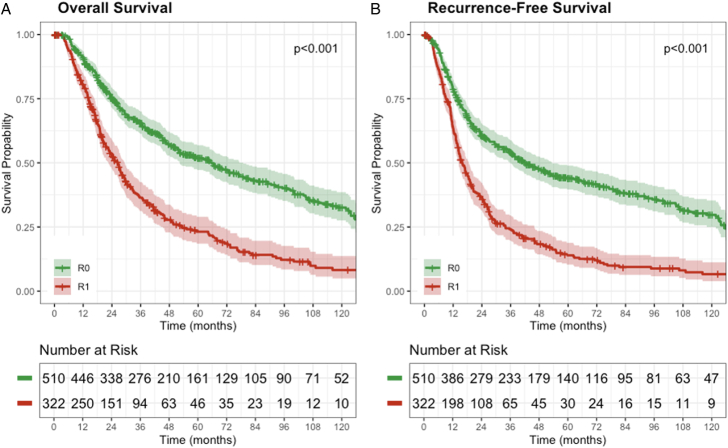
Kaplan-Meier survival curves for overall (A) and recurrence-free (B) survival stratified by margin status.

The median follow-up for surviving patients was 45.3 months (IQR: 22.0–81.0). Median OS was significantly longer in patients with an R0 margin [65.8 mo (95% CI: 52.3–78.8)] compared with those with an R1 margin [26.3 mo (95%CI: 22.6–29.3, *P*<0.001, Fig. 1A]. Similarly, the median RFS was significantly longer in patients with an R0 margin [42.6 mo (95% CI: 35.8–53.2)] compared with those with an R1 margin [15.8 mo (95% CI: 14.3–18.5, *P*<0.001, Fig. 1B]. Alike, local-specific progression was significantly more common in patients with an R1 margin compared with those with an R0 margin (34% vs 19%, *P*<0.001). When multiple margins were involved by invasive cancer, the rate of local recurrence was 55% compared with 32% after an R1 uncinate margin (*P*=0.080) and 23% after an R1 neck margin (*P*=0.023, Supplemental Table 1, Supplemental Digital Content 1, http://links.lww.com/SLA/F302). R1 margin location and corresponding recurrence rates and overall survival are summarized in Supplemental Table 1, Supplemental Digital Content 1, http://links.lww.com/SLA/F302. Comparisons between direct and indirect R1 margins are described in Supplemental Text 2, Supplemental Digital Content 1, http://links.lww.com/SLA/F302 and Supplemental Figure 1, Supplemental Digital Content 1, http://links.lww.com/SLA/F302.

### Longitudinal Outcomes Based on Isolated Pancreatic Neck Margin

**FIGURE 2:**
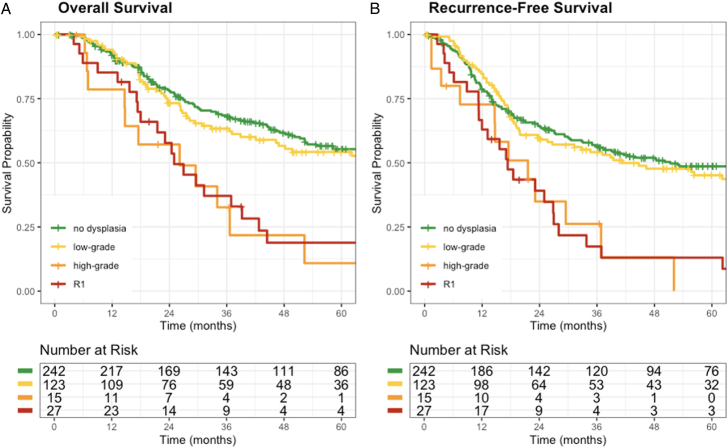
Kaplan-Meier survival curves for overall (A) and recurrence-free (B) survival stratified by definitive margin status at the pancreatic neck.

Comparing survival among patients with definitive pancreatic neck pathology and where the neck margin was the worst pathologic margin (Fig. 2A), those without any dysplasia (true R0) had similar OS to those with LGD (mOS: 78.8 vs 66.8 mo, *P*=0.344). However, those with HGD [mOS: 26.1 mo (95% CI: 14.7–NR)] had worse survival than those with true R0 (*P*<0.001) and LGD (*P*=0.001). Similarly, those with an R1 margin [mOS: 25 mo (95% CI: 21.5–42.7)] also had poorer survival than those with true R0 (*P*<0.001) and LGD (*P*=0.005). Patients with HGD or R1 at the margin had similar survival (*P*=0.471). A similar trend was observed for RFS in these patients (Fig. 2B).

### Impact of Pancreatic Neck Margin and Dysplasia on Surgical Practice

Of the 510 patients with an R0 margin, 412 had detailed neck margin pathology documented. Of these, 275 (67%) had normal pancreatic epithelium at the pancreatic neck margin, while 121 (29%) and 16 (4%) had LGD and HGD present.

Of the 322 patients with any R1 margin location (including those with multiple sites of R1 margins on final pathology), 218 had detailed neck margin pathology documented. Of these, 82 (38%), 96 (44%), 6 (3%), and 34 (16%) had normal pancreatic epithelium, LGD, HGD, or invasive cancer within 1 mm of the final pancreatic neck margin.

After excluding patients who underwent a preoperatively planned total pancreatectomy, as well as those with missing data regarding frozen sections, a total of 433 patients were found to have undergone intraoperative frozen section assessment of the pancreatic neck. Of these, 145 (33%) had a negative frozen section with normal pancreatic epithelium and did not undergo further reresection. IPMN with low-grade dysplasia at the margin was observed in 178 (41%) of frozen sections, while high-grade dysplasia was reported in 15 (3%) patients. Invasive cancer was noted in the remaining 95 (22%) patients.

Of these 433 patients, 133 patients (31%) underwent further reresection [133/288 (46%) of patients with dysplasia or invasive cancer at the neck margin]. Of these 133 patients, 27 patients (20%) were reresected for LGD, 13 patients (10%) were reresected for HGD, 91 patients (68%) were reresected for invasive cancer, while the remaining 2 patients (2%) underwent reresection during intraoperative observation of multifocal IPMN (Supplemental Table 2, Supplemental Digital Content 1, http://links.lww.com/SLA/F302). Of these reresections, in 74 patients (56%) an intraoperative decision to perform a total pancreatectomy was made, while a decision to perform extended partial pancreatectomy was made intraoperatively for the remaining 59 patients (44%).

Of the 27 patients with intraoperative LGD at the neck margin, 5 patients (19%) underwent total pancreatectomy for multiple reasons including LGD at the margin and multifocal IPMN, while of the remaining 22 patients (81%) underwent additional partial parenchymal reresection. In those who underwent reresection, 19 patients (86%) still had LGD, and 3 patients (14%) were reresected to normal pancreatic epithelium (Supplement Table 2, Supplemental Digital Content 1, http://links.lww.com/SLA/F302).

Of the 13 patients with intraoperative HGD at the neck margin, 4 patients (31%) underwent total pancreatectomy, while of the remaining nine patients (69%) that underwent an additional partial parenchymal reresection, 1 patient (11%) still had HGD on final pathology, 2 patients (22%) had LGD, and 6 patients (67%) were reresected to normal pancreatic epithelium (Supplemental Table 2, Supplemental Digital Content 1, http://links.lww.com/SLA/F302).

Of the 91 patients with intraoperative invasive carcinoma at the neck margin, 63 patients (69%) underwent total pancreatectomy, while of the remaining 28 patients (31%) who underwent an additional partial parenchymal reresection, final pathology remained invasive carcinoma in 2 patients (7%), while HGD, LGD, and normal pancreatic epithelium were found in 2 (7%), 4 (14%), and 20 (71%) patients (Supplemental Table 2, Supplemental Digital Content 1, http://links.lww.com/SLA/F302).

### Conversion of High-risk to Low-risk Versus High-risk Neck Margin

**FIGURE 3:**
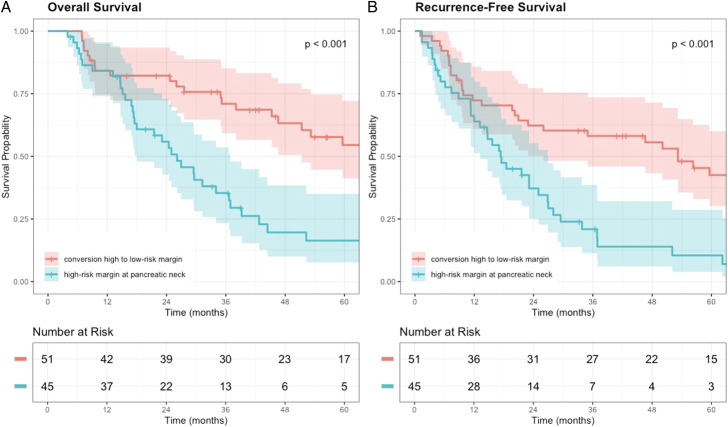
Kaplan-Meier survival curves for overall (A) and recurrence-free (B) survival stratified by reresection of pancreatic neck margin with conversion of high-risk margin to low-risk margin versus definitive high-risk neck margin.

Those who underwent reresection of an intraoperative high-risk neck margin and conversion to a low-risk neck margin on final pathology (N=51, only patients with isolated pancreatic neck margin involvement) had superior associated OS [mOS: 76.9 mo (95% CI: 51.3–126.8)] than those with a high-risk neck margin (N=45) on final pathology [mOS: 26.1 mo (95% CI: 18.0–36.9), *P*<0.001, Fig. 3A]. A similar observation was seen comparing RFS (mOS: 53.2 mo vs 17.3 mo, *P*<0.001, Fig. 3B) and time to local-specific recurrence (mOS: NR vs 36.9 mo, *P*<0.001). Comparing these 2 groups, patients had similar clinicopathologic characteristics including age (*P*=0.736), year of operation (*P*=0.635), sex (*P*=0.858), CA19-9 (*P*=0.439), tubular histology (*P*=0.709), and receipt of adjuvant chemotherapy (*P*=0.391). However, those patients with a high-risk margin on final pathology had more advanced T-stage (*P*=0.020) and N-stage (*P*=0.004). After multivariable analysis adjusting for T and N-stage, conversion of a high-risk to a low-risk neck margin remained independently associated with improved OS.

Even when including all patients (irrespective of alternative margin site status), patients with intraoperative transected neck margin conversion from high to low-risk, had an associated superior OS [mOS: 35.3 mo (95% CI: 18.0–34.6)] than those with a high-risk neck margin on final pathology [mOS: 27.0 mo (95% CI: 26.0–59.6), *P*=0.014].

### Multivariable Cox-regression for OS and RFS for High-risk Neck Margin in All Patients

**FIGURE 4:**
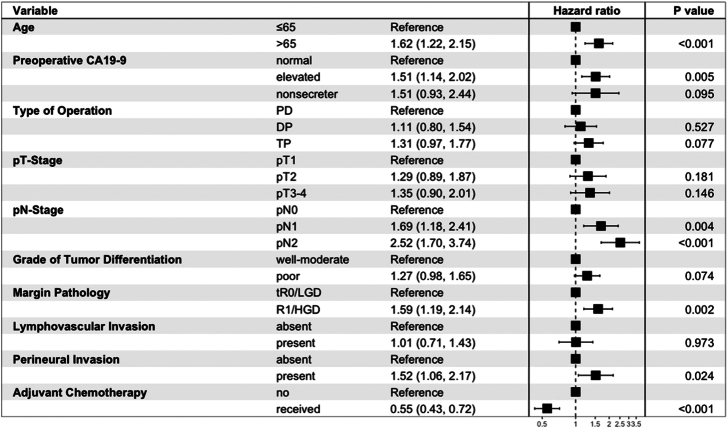
Multivariable Cox-regression for overall survival.

High-risk margin pathology was independently associated with worse OS (HR: 1.59, 95% CI: 1.19–2.14) as compared with a low-risk margin (Fig. 4). Similar effects were observed for the multivariable Cox-regression analysis on RFS with high-risk margins being associated with higher hazards (HR: 1.43, 95% CI: 1.09–1.87) compared with low-risk margins (Supplemental Figure 2, Supplemental Digital Content 1, http://links.lww.com/SLA/F302).

## DISCUSSION

This largest international multicenter study investigating the role of margin status in IPMN-derived PDAC observed that patients with normal epithelium (true R0) or low-grade dysplasia at the neck have similar outcomes, while high-grade dysplasia or invasive cancer (ie, high-risk margins) are both associated with worse overall and RFS. Reresection of the pancreatic neck after intraoperative assessment is associated with improved outcomes when a high-risk margin is successfully transformed to a low-risk margin. These findings thus support the importance of intraoperative frozen section for decision-making and reresection of high-risk neck margins, when possible, to improve survival and recurrence outcomes even in the setting of invasive cancer arising from IPMN.

The updated Kyoto guidelines in 2023 recommend that when a frozen section of a transected margin indicates invasive cancer or HGD, additional reresection is recommended.^17^ Alternatively, some studies have contended that the presence of invasive cancer in the resected specimen dictates outcomes and thus the presence of HGD at the margin does not mandate further resection.^16,19^ In addition, studies have reported favorable outcomes even in patients with HGD at the margin.^24–26^ However, our cohort including only patients with IPMN-derived PDAC does not support latter hypothesis or finding, as patients with HGD or IC at the neck margin had similarly dismal outcomes and conversion of a high-risk pancreatic neck margin was associated with superior OS and RFS. These findings underscore the importance of intraoperative frozen sections to guide intraoperative decisions for margin handling including a possible reresection and conversion of high-risk margins to low risk margins, thus supporting the expert recommendations.^17,27^ Interestingly, when interpreting the Kaplan-Meier curves in Figure 3, the conversion group and high-risk margin group demonstrate similar outcomes within the first year after surgery. However, the survival benefit associated with high-risk margin conversion becomes evident after 12 months which coincides with a delayed time to local recurrence compared with liver recurrence observed in PDAC.^5^ Therefore, a potential for decreased local recurrence after high-risk margin conversion may partially explain the belated associated benefit in RFS.

The presence of LGD dysplasia at the margin has been studied with mixed results, resulting in the current expert recommendation being that there is insufficient data to support further resection.^18,28–30^ Herein, we found that normal epithelium at the margin had similar outcomes to LGD at the margin. Thus, these findings again support current guideline recommendations that the presence of normal epithelium or LGD at the transected margin does not necessitate further reresection in patients with IPMN-derived PDAC. These findings are further backed by studies reporting that postoperative recurrence in patients with LGD at the margin typically arises from an independent neoplasm.^30,31^

The prognostic difference of direct versus indirect R1 margins has recently been put into question with a study by Strobel et al demonstrating that R1-substaging is associated with a difference in survival in PDAC.^32^ Interestingly, in our cohort we did not observe a significant difference in OS and RFS by R1 substaging. However, patients with a direct R1 margin were associated with a significantly faster time to local progression. The lack of significant impact of subclassifying R1 margins on survival supports unifying direct and indirect margins; however, the presence of invasive cancer directly involving the margin with its association to shorter time to local progression may have implications for surveillance. In addition, this correlation of local recurrence to increasing margin involvement was also seen for recurrence sites with multiple margins being associated with more local recurrences as compared with the most frequently encountered single sites of margin positivity.

The routine practice of intraoperative frozen sectioning for pancreatic cancer remains controversial.^33^ This is true for the more prevalent PanIN-derived PDAC, a biologically and clinically distinct entity, as well as for IPMN-derived pancreatic cancer.^34–37^ Surgeons who do not routinely practice intraoperative frozen sectioning argue that a positive microscopic margin is a surrogate of poor biology and that these patients can also have other R1 margins at nonmodifiable sites. In this setting, reresection may not portend survival benefit and therefore the associated postoperative risks of an extended or total pancreatectomy outweigh the benefit derived from reresection. Alternatively, advocates of intraoperative frozen sectioning argue that this practice guides the extent of resection and increases the rate of complete oncologic resections, which is paramount in improving survival outcomes. In line with our findings in IPMNs, a recent meta-analysis by Crippa et al^13^ found that conversion of an R1 neck margin to R0 was associated with better survival than an R1 margin on final pathology in PanIN-derived PDAC. One step further, we found that patients with HGD at the transected margin have similar outcomes to patients with invasive cancer, and thus deemed both cohorts as high-risk. Future studies in a larger cohort of patients should validate this finding and assess if a graded associated benefit may exist, where patients with invasive cancer benefit more from reresection than those with HGD. Given the relatively indolent systemic biology of IPMN-derived PDAC, as compared with its counterpart, one could argue that a more aggressive approach to local therapy, ie, extent of resection could have an even more profound impact on survival.

Several limitations in the present study should be acknowledged. First, although a large proportion of patients had details documented on intraoperative frozen section, a fraction did not. Moreover, location of R1 margin status was not always routinely documented in pathology reports. Prospectively designed studies are needed to validate our observations. Next, the role of intraoperative pancreatoscopy to assess for skip lesions is gaining momentum and may further inform surgical decisions beyond the pancreatic neck.^38^ Ongoing trials are investigating its utility, which may serve as an adjunct to frozen sections to inform intraoperative decision-making. The presence of a field defect in IPMN is widely supported and de novo lesions may have impacted our findings.^39^ Finally, pathologic reporting practices differ by institution or year of examination, and unification of international guidelines are ongoing and urgently needed.

In conclusion, this international multicenter study investigating the impact of resection margin status in IPMN-derived PDAC demonstrated that patients resected with R1 margins have an association with worse recurrence-free and overall survival compared with patients with an R0 resection. While normal epithelium and low-grade dysplasia at the neck have similar outcomes, pancreatic neck margins with high-grade dysplasia or invasive cancer are high-risk margins associated with poorer longitudinal outcomes. Reresection of these high-risk margins is associated with improved outcomes when successful conversion into a low-risk margin was obtained. These findings underscore the importance of intraoperative frozen section for intraoperative decision-making on margin handling. If feasible, reresection of high-risk neck margins, should be performed to improve survival and recurrence outcomes even in patients with IPMN-derived pancreatic cancer.

## Supplementary Material
