# Supplementary material for: Informing Decision-making for Transected Margin Reresection in Intraductal Papillary Mucinous Neoplasm-derived Pancreatic Cancer: An International Multicenter Study
Source: Ann Surg. 2024 Sep 12;283(6):1051–9. doi: 10.1097/SLA.0000000000006532 (PMC13218440; doi:10.1097/SLA.0000000000006532)

**SUPPLEMENT**

**Supplement Text 1.**

*Intraoperative and Postoperative Outcomes for R1 resection Compared to R0*

American Anesthesiologist Association score (p=0.150), age (p=0.200), year of surgery (0.740), and type of operation (p=0.082) were similar between the R1 and R0 groups. R1 margin status was associated with more vascular resection (32% vs. 9%, p<0.001), blood loss (median 600 vs. 500 ml, p=0.001), postoperative complications (60% vs. 51%, p=0.007), reoperation (9% vs. 5%, 0=0.029), and median length of stay (13 vs. 12 days, p=0.001). Operative duration was similar between the two groups (315 vs. 320 minutes, p=0.465). Postoperatively, patients with an R1 margin were more likely to receive adjuvant chemotherapy (65% vs. 56%, p=0.006) but equal median number of completed cycles were observed in those receiving adjuvant chemotherapy (both groups median 6 cycles, IQR:4-6, p=0.142).

**Supplement Text 2.**

*Longitudinal Outcomes Comparing Indirect and Direct R1 Margins*

Patients with an R0 resection had an associated superior OS [mOS: 65.8 months (95%CI: 52.3-78.8)] compared to those indR [mOS: 27.5 months (95%CI: 22.6-34.6)] and dR1 [mOS: 25.2 months (95%CI: 20.2-33.1)] (p<0.001). However, no significant difference in OS was observed between patients indR1 and dR1 (Supplement Figure 1A, p=0.540). A similar trend was observed for RFS in these patients (Supplement Figure 1B). Interestingly, time to local-specific progression was favorable for indR1compared to dR1 (p=0.029, Supplement Figure 1C).

**Supplement Table 1.** Location of R1 margin site on final pathology with corresponding local recurrence rates and median overall survival.

| **Site of R1 Margin** | **N (%)** | **Local Recurrence (%)**** | **Median Overall Survival** | **95% Confidence Interval** |
| --- | --- | --- | --- | --- |
| Uncinate* | 53 (28) | 13 (32) | 25.2 | 15.3-49.0 |
| Posterior Surface* | 50 (26) | 14 (39) | 24.9 | 19.0-35.0 |
| Pancreatic Neck* | 31 (16) | 7 (23) | 25.0 | 18.0-42.7 |
| Vascular Groove* | 18 (10) | 8 (47) | 28.3 | 20.7-NR |
| Anterior Surface* | 6 (3) | 2 (40) | 24.2 | 18.4-NR |
| Enteric* | 3 (2) | 1 (50) | 33.1 | 29.0-NR |
| Bile Duct* | 2 (1) | 0 (0) | 25.6 | 4.9-NR |
| Multiple Sites | 27 (14) | 11 (55) | 18.0 | 12.1-53.1 |
| Not specified | 132 | 25 (30) | 27.8 | 21.5-36.6 |

***isolated sites of R1 margin**

****missing values on local recurrence in 38 patients with specific recurrence sites**

**Supplement Table 2.** Reason, handling, and final pathology of transected pancreatic neck margin after intraoperative frozen sectioning.

| Intraoperative Reason for Reresection | N (%) | Partial Pancreatectomy  N (%) | Final Pathology  N (%) | Intraoperative Total Pancreatectomy  N (%) |
| --- | --- | --- | --- | --- |
| Low-grade dysplasia | 27 (20) | 22 (81) | True R0: 3 (14) | 5 (19) |
|  |  |  | LGD: 19 (86) |  |
| High-grade dysplasia | 13 (10) | 9 (69) | True R0: 6 (67) | 4 (31) |
|  |  |  | LGD: 2 (22) |  |
|  |  |  | HGD: 1 (11) |  |
| Invasive cancer | 91 (68) | 28 (31) | True R0: 20 (71) | 63 (69) |
|  |  |  | LGD: 4 (14) |  |
|  |  |  | HGD: 2 (7) |  |
|  |  |  | R1: 2 (7) |  |
| Multifocal disease | 2 (2) | 0 (0) | - | 2 (100) |

**Supplement Figure 1.** Kaplan-Meier survival curves for overall survival (A) recurrence-free survival (B) and time to local progression (C) stratified by R0, direct R1, and indirect R1 margins


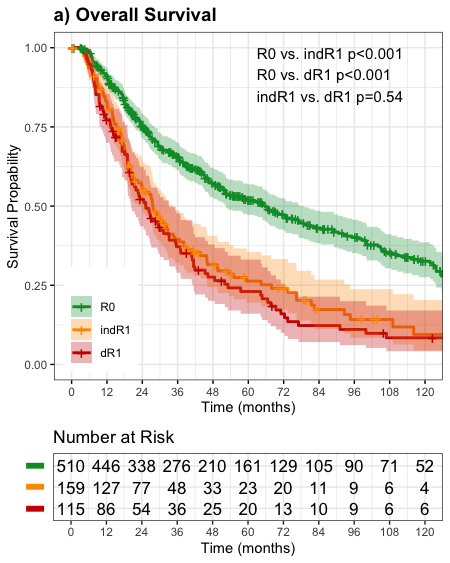

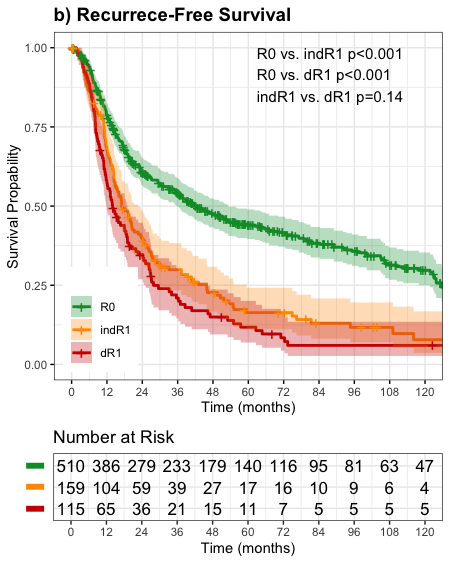


**
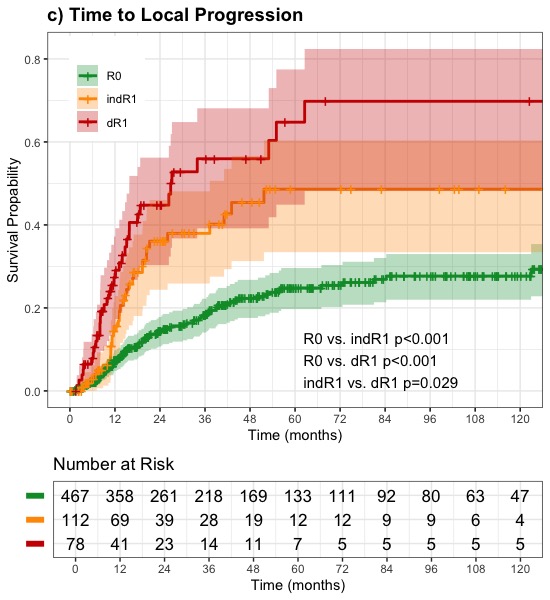
**

**Supplement Figure 2.** Multivariable Cox-Regression for Recurrence-Free Survival


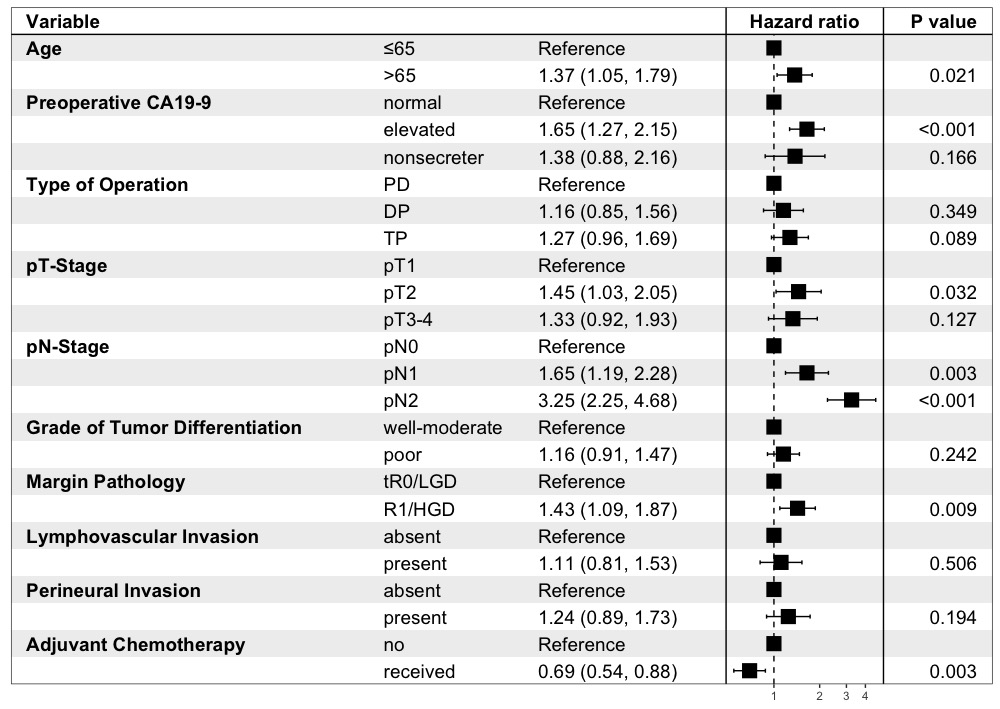

Supplement: Supplementary file 1 [file sla-283-1051-s001.docx]
